# Age-Dependent Decline in Cardiac Function in Guanidinoacetate-*N*-Methyltransferase Knockout Mice

**DOI:** 10.3389/fphys.2019.01535

**Published:** 2020-01-21

**Authors:** Dunja Aksentijević, Sevasti Zervou, Thomas R. Eykyn, Debra J. McAndrew, Julie Wallis, Jurgen E. Schneider, Stefan Neubauer, Craig A. Lygate

**Affiliations:** ^1^Radcliffe Department of Medicine, Division of Cardiovascular Medicine and Wellcome Centre for Human Genetics, University of Oxford, Oxford, United Kingdom; ^2^Department of Imaging Chemistry and Biology, School of Biomedical Engineering and Imaging Sciences, King’s College London, St Thomas’ Hospital, London, United Kingdom; ^3^Experimental and Preclinical Imaging Centre, Leeds Institute of Cardiovascular and Metabolic Medicine, University of Leeds, Leeds, United Kingdom

**Keywords:** cardiac energetics, creatine, energy metabolism, mitochondrial respiration, ventricular function

## Abstract

**Aim:**

Guanidinoacetate *N*-methyltransferase (GAMT) is the second essential enzyme in creatine (Cr) biosynthesis. Short-term Cr deficiency is metabolically well tolerated as GAMT^–/–^ mice exhibit normal exercise capacity and response to ischemic heart failure. However, we hypothesized long-term consequences of Cr deficiency and/or accumulation of the Cr precursor guanidinoacetate (GA).

**Methods:**

Cardiac function and metabolic profile were studied in GAMT^–/–^ mice >1 year.

**Results:**

*In vivo* LV catheterization revealed lower heart rate and developed pressure in aging GAMT^–/–^ but normal lung weight and survival versus age-matched controls. Electron microscopy indicated reduced mitochondrial volume density in GAMT^–/–^ hearts (*P* < 0.001), corroborated by lower mtDNA copy number (*P* < 0.004), and citrate synthase activity (*P* < 0.05), however, without impaired mitochondrial respiration. Furthermore, myocardial energy stores and key ATP homeostatic enzymes were barely altered, while pathology was unrelated to oxidative stress since superoxide production and protein carbonylation were unaffected. Gene expression of PGC-1α was 2.5-fold higher in GAMT^–/–^ hearts while downstream genes were not activated, implicating a dysfunction in mitochondrial biogenesis signaling. This was normalized by 10 days of dietary Cr supplementation, as were all *in vivo* functional parameters, however, it was not possible to differentiate whether relief from Cr deficiency or GA toxicity was causative.

**Conclusion:**

Long-term Cr deficiency in GAMT^–/–^ mice reduces mitochondrial volume without affecting respiratory function, most likely due to impaired biogenesis. This is associated with hemodynamic changes without evidence of heart failure, which may represent an acceptable functional compromise in return for reduced energy demand in aging mice.

## Introduction

Creatine (Cr) is a nitrogenous organic acid derived from glycine, L-arginine and S-adenosyl-L methionine in a two-step reaction catalyzed by enzymes L-arginine:glycine amidino transferase (AGAT) in the kidney and guanidinoacetate N-methyltransferase (GAMT) in the liver (Lygate et al., 2013). In organs with high energy demand such as the heart, creatine is taken up by cardiomyocytes via a specific membrane transporter (CrT), whereupon it is interconverted to phosphocreatine (PCr), under the control of creatine kinase (CK). This system functions as a short-term energy buffer, maintains thermodynamically favorable levels of local reactants, and shuttles high-energy phosphates from mitochondria to sites of utilization such as the myofibril (Wyss and Kaddurah-Daouk, 2000; Ingwall and Weiss, 2004; Schlattner et al., 2006; Brosnan and Brosnan, 2007). An impaired PCr/CK system has been implicated in the pathophysiology of heart failure, with key components consistently down-regulated in both human and animal models of heart failure, regardless of etiology (Ingwall and Weiss, 2004; Ventura-Clapier et al., 2004; Lygate et al., 2007).

However, whether such changes have a causative role for the development of contractile dysfunction has been a matter of debate (Neubauer, 2007). One way to address this question is to determine whether loss-of-function models recapitulate a heart failure phenotype. Feeding the creatine analog β-guanidinopropionic acid (β-GPA) to rats has a profound effect on energetic parameters including mitochondria, but the effect on contractile function has been equivocal (e.g., Shoubridge et al., 1985; Kapelko et al., 1988; Mekhfi et al., 1990; Neubauer et al., 1999). However, creatine analogs have many limitations, such as pharmacological off-target effects, and slow and incomplete creatine depletion. Genetic loss-of-function mouse models circumvent these limitations and provide a unique approach to assess the functional significance of the PCr/CK system.

The creatine biosynthetic enzymes are not expressed in the heart and therefore require global knockout. AGAT^–/–^ mice have undetectable levels of creatine and PCr with impaired *in vivo* contraction and relaxation at rest. However, most of this phenotype was attributable to homoarginine rather than creatine deficiency (Faller et al., 2018). GAMT knockout mice (GAMT^–/–^) fed a creatine-free diet have a chronic, and absolute, deficiency of creatine and PCr (Schmidt et al., 2004; Lygate et al., 2013), although they accumulate the creatine precursor guanidinoacetate (GA), and phospho-guanidinoacetate (PGA). GAMT^–/–^ mice have a whole-body phenotype of greatly reduced body weight, due to both lower fat and muscle mass, which can also confound interpretation. We have previously shown that GAMT^–/–^ mice have normal LV ejection fraction up to 1 year of age (Schneider et al., 2008), but with mildly reduced systolic pressure development (Ten Hove et al., 2005b). Under conditions of maximal β-adrenergic stimulation, contractile reserve is reduced and GAMT^–/–^ mice show impaired functional recovery from ischemia, in keeping with the prevailing view that the PCr/CK system is particularly important under conditions of high workload and acute stress (Wyss and Kaddurah-Daouk, 2000; Ingwall and Weiss, 2004). However, when GAMT^–/–^ mice were subject to chronic myocardial infarction, these defects were not sufficient to negatively impact on survival, *in vivo* cardiac function, or LV structural remodeling, suggesting that loss of creatine does not exacerbate contractile dysfunction in heart failure (Lygate et al., 2013).

The current study was borne out of the observation that LV hemodynamic parameters in GAMT^–/–^ declined beyond 1 year of age compared to our historical data sets. We hypothesized that unidentified compensatory adaptations may allow young adult GAMT^–/–^ hearts to compensate for chronic creatine deficiency, but that these are not sustainable in the long-term. We therefore sought to identify whether metabolic (mal)adaptations develop in the aging (>1 year) GAMT^–/–^ mice. Herein, we show for the first time that prolonged and chronic creatine deficiency results in reduced mitochondrial volume density and a shift in adaptations from energy production to energy saving in older GAMT^–/–^ mice concomitant with a decline in cardiac function. Our study underscores the plasticity and connectivity of energy generating pathways and the need for compensatory strategies to adapt in response to the aging heart.

## Results

### *In vivo* Cardiac Function Declines With Age in GAMT^–/–^

**FIGURE 1:**
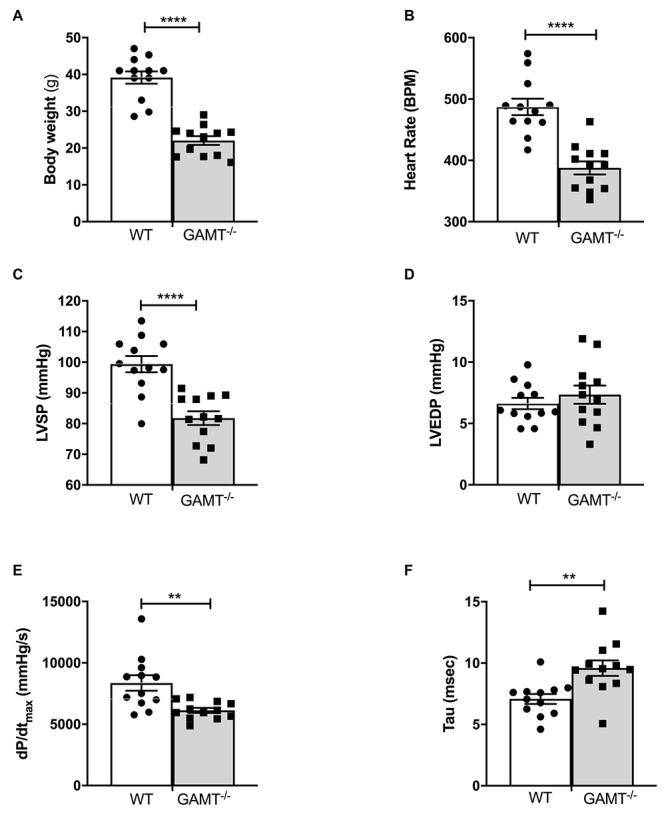
*In vivo* left ventricular hemodynamic function in WT and GAMT^–/–^ mice at 18 months of age **(A)** GAMT^–/–^ mice have lower body weight throughout their life. **(B)** Heart rate, **(C)** LV systolic pressure (LVSP), **(D)** LV end-diastolic pressure (LVEDP), **(E)** maximal rate of pressure rise (d*P*/d*t*_*max*_) as a measure of contractility, and **(F)** time constant of isovolumetric relaxation (tau). *N* = 12 (5F/7M) per genotype, ^∗∗^denotes *P* < 0.01, ^∗∗∗∗^*P* < 0.0001 for WT versus KO at the same age by unpaired *t*-test **(A–D,F)** and Welch’s *t*-test **(E)**. All values are mean ± SEM.

As expected, body weight was very low in 18 month old GAMT^–/–^ mice (Figure 1A), since creatine deficiency results in low skeletal muscle mass and body fat. *In vivo* LV catheterization demonstrated a hemodynamic profile in GAMT^–/–^ consisting of lower heart rate and LV systolic pressure, reduced pressure generation (d*P*/d*t*_*max*_) with prolonged tau of isovolumetric relaxation (Figure 1). Absolute values for these parameters were lower than observed in younger animals collected under identical experimental conditions in our laboratory [previously published in Ten Hove et al. (2005b) and Lygate et al. (2013)], whereas WT values were comparable across studies. It is notable that, LV end-diastolic pressure did not change, nor was there evidence of pulmonary congestion or LV hypertrophy (lung, LV, and RV weights were not elevated; Table 1). This suggests that the decline in function is not associated with a heart failure phenotype and hence we do not observe concomitant mortality at this age (Supplementary Figure 1). This suggests that the decline in function is not associated with a heart failure phenotype.

**TABLE 1:** Morphometric parameters and metabolic analysis of aging GAMT^–/–^ mice.

	**WT**	**GAMT^–/–^**
**Morphology**		
Age (weeks)	76 ± 0.9	76 ± 3.2
LV weight (mg)	106.1 ± 4.4	87.0 ± 5^∗^
RV weight (mg)	27.2 ± 1	23.4 ± 2
Lung weight (mg)	163.1 ± 5	153.4 ± 1
Tibial length (mm)	18.7 ± 0.1	18.2 ± 0.1^∗∗^
LV/Tibia Length (mg/mm)	5.6 ± 0.2	4.7 ± 0.3^∗^
LV/Body Weight (mg/g)	2.6 ± 0.1	3.5 ± 0.1^*⁣**^
**Myocardial energetic profile**		
TAN Pool (nmol/mg protein)	31.1 ± 2.7	40.1 ± 4.9
Creatine (μmol/g whw)	12.1 ± 0.6	0.07 ± 0.01^*⁣**^
Phosphocreatine (μmol/g whw)	0.8 ± 0.05	0.07 ± 0.02^*⁣**^
Guanidinoacetate (μmol/g whw)	0.3 ± 0.05	7.4 ± 0.8^*⁣**^
Phosphoguandinoacetate (μmol/g whw)	0.2 ± 0.12	3.5 ± 0.53^*⁣**^
Triacylglycerol (mM/whw)	0.05 ± 0.006	0.03 ± 0.002^∗^
Glycogen (glycolsyl units/g whw)	4.3 ± 0.5	4.1 ± 0.83
**Plasma metabolites**		
Free Fatty Acids (μmol/l)	541.7 ± 74.8	517.1 ± 57.86
3-hydroxybutyrate (μmol/l)	247.3 ± 32.05	249.6 ± 15.7
Cholesterol (mmol/l)	3.4 ± 0.6	2.0 ± 0.1^∗^
Triglycerides (mmol/l)	1.6 ± 0.2	0.8 ± 0.1^∗∗^
High Density Lipoprotein (mmol/l)	1.6 ± 0.3	0.9 ± 0.2
Low Density Lipoprotein (mmol/l)	1.1 ± 0.3	0.8 ± 0.1
Lactate (mmol/l)	10.2 ± 5.2	5.2 ± 2.6
Total Creatine Kinase (IU/l)	3404 ± 1275	936.5 ± 276
Lactate Dehydrogenase (IU/l)	714.0 ± 71.1	871.2 ± 240

*Whw – wet heart weight, LV – left ventricle, RV – right ventricle, TAN – total adenine nucleotide pool (ATP + ADP + AMP). Morphological parameters (*n* = 12/group 5F/7M), energetic profile (*n* = 4–5/group 3F/2M), plasma metabolites (*n* = 3–5/group 2F/3M). Concentrations of creatine, PCr, guanidinoacetate (GA), and phospho-guanidinoacetate (P-GA) measured by ^1^H-NMR spectroscopy. Total adenine nucleotide pool (TAN pool = ADP + AMP + ATP) assessed by HPLC. All values are mean ± SEM. Comparisons were made by Student’s *t*-test. ^∗^*P* < 0.05, ^∗∗^*P* < 0.01, ^∗∗∗^*P* < 0.001.*

### Metabolic Profile in GAMT^–/–^ Hearts

**FIGURE 2:**
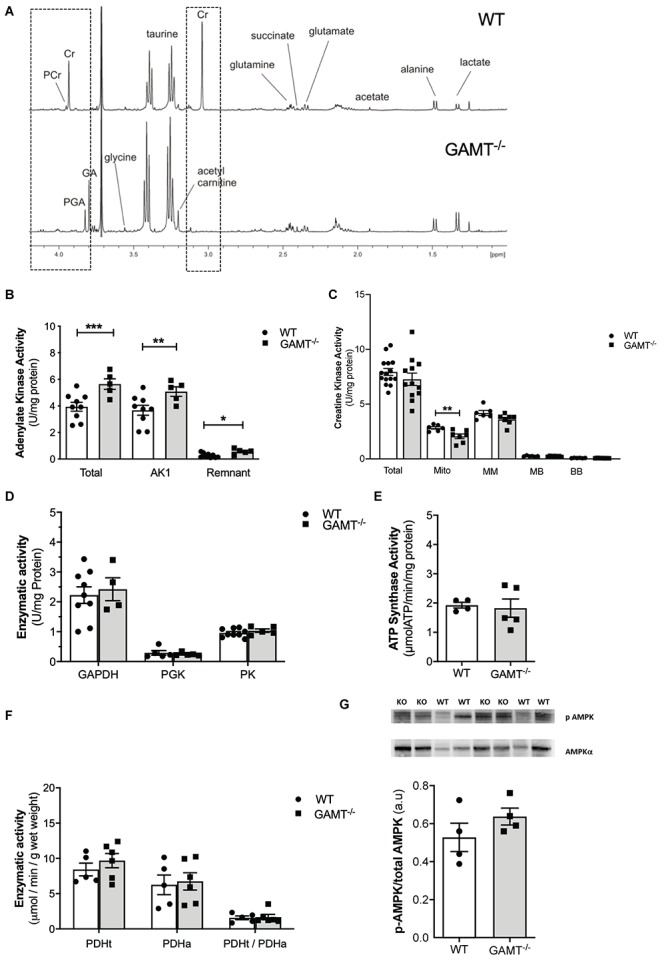
Cardiac energetic profile in >1 year GAMT^–/–^ and wild type mice. **(A)** Representative ^1^H-NMR spectra showing metabolomic profile of WT and GAMT^–/–^ heart. Highlighted (dotted line) is the complete absence of PCr and Cr but presence of P-GA and GA in GAMT^–/–^ sample versus WT. Enzyme activities for **(B)** adenylate kinase (AK) (WT *n* = 9 5F/4M, GAMT^–/–^ *n* = 5 2F/3M), **(C)** creatine kinase – Total (WT *n* = 15 GAMT^–/–^ *n* = 12), mitochondrial CK (Mito), MM, MB, and BB isoforms (WT *n* = 6 GAMT^–/–^ *n* = 7), **(D)** glycolytic enzymes glycerlaldehyde-3-phosphate dehydrogenase (GAPDH) (WT *n* = 9 5F/4M GAMT^–/–^ *n* = 4 2F/2M), 3-phosphoglycerate kinase (PGK) (WT *n* = 5 2F/3M GAMT^–/–^ *n* = 5 2F/3M), pyruvate kinase (PK) (WT *n* = 9 5F/4M GAMT^–/–^ *n* = 5 2F/3M), **(E)** F_1_F_0_ ATP Synthase (mitochondrial electron transport chain complex V) (WT *n* = 4 1F/3M GAMT^–/–^ *n* = 5 3F/2M), **(F)** pyruvate dehydrogenase total (PDHt), active (PDHa), and ratio of total to active (PDHt/PDHa) as an indicator of the extent of enzyme complex activation (*n* = 5/group 3F/2M), and **(G)** AMPK protein expression (*n* = 4/group) ^∗^Denotes *P* < 0.05, ^∗∗^*P* < 0.01, ^∗∗∗^*P* < 0.001 for GAMT^–/–^ versus wild type by unpaired *t*-test. Mouse mean age-84 weeks. All values are mean ± SEM.

^1^H NMRS metabolite analysis of extracted aging GAMT^–/–^ heart tissue confirmed complete depletion of creatine and PCr and the resultant accumulation of GA and PGA (Table 1 and Figure 2A). In the absence of a fully functioning PCr/CK system, we assessed a range of alternative phosphotransfer enzymes, energy stores and metabolic regulators. Young GAMT^–/–^ hearts exhibit reduced CK activity, normal adenylate kinase (AK) activity and elevated activities for glyceraldehyde-3-phosphate dehydrogenase (GAPDH) and pyruvate kinase (PK) (Lygate et al., 2013).

In contrast, aging GAMT^–/–^ hearts had 43% higher AK activity compared to age-matched WT (Figure 2B; *P* < 0.008) but the rest of metabolic profile differences were normalized: CK, GAPDH, PK activities (Figures 2C,D), glycogen content (Table 1 and Supplementary Table 1). Similarly, the maximal activity of F_1_-ATP synthase was elevated in hearts from young GAMT^–/–^ mice (Supplementary Table 1), but normalized in the aging animals (Figure 2E).

Activity of pyruvate dehydrogenase (PDH) was unchanged irrespective of age (Supplementary Table 1 and Figure 2F), suggesting that oxidative glycolytic contribution to the Krebs cycle was unaltered. The total adenine nucleotide (TAN) pool did not differ between GAMT^–/–^ and WT regardless of age (Table 1 and Supplementary Table 1), nor was there activation of the AMP-activated protein kinase (AMPK), as measured by total AMPKα expression (normalized to total protein WT 0.51 ± 0.07 vs. GAMT^–/–^ 0.47 ± 0.01, *P* = 0.26) or in phosphorylation status (Figure 2G). Since this is a whole-body knockout, we measured circulating metabolites: lactate, free fatty acids, 3-hydroxybutyrate, high density lipoprotein and markers of muscle damage (lactate dehydrogenase, CK) were not significantly different between genotypes (Table 1). However, both serum and myocardial triacylglycerol content was lower in GAMT^–/–^ vs. WT irrespective of age (Table 1 and Supplementary Table 1), probably reflecting lower total body fat in knockout mice (Lygate et al., 2013).

### Impact of GAMT^–/–^ on Mitochondrial Organization and Function

**FIGURE 3:**
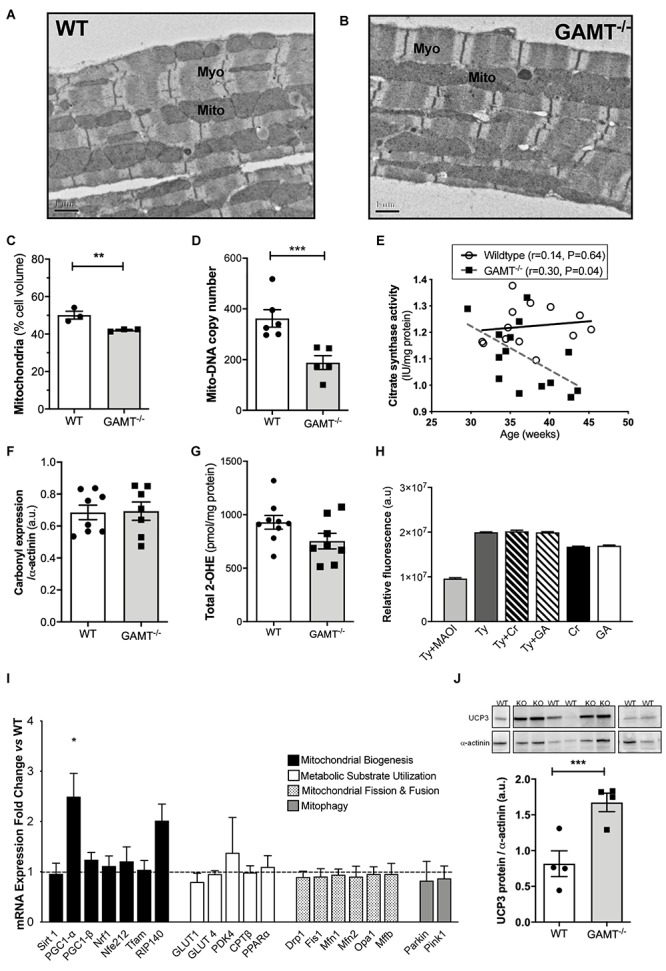
Mitochondrial phenotype develops with age in GAMT^–/–^ mice **(A,B)** Representative electron micrographs at 50 week age; 10000× magnification. **(C)** Stereological analysis shows lower mitochondrial volume density in GAMT^–/–^ compared to WT (*n* = 3). **(D)** Mitochondrial DNA copy number in a separate group of mice (WT *n* = 6, GAMT^–/–^ *n* = 5). **(E)** Citrate synthase activity inversely correlates with age in KO, but not in WT mice (*n* = 14/group 6F/8M). **(F,G)** Protein carbonylation in LV homogenates from >1 year old mice was not different (*n* = 7–8) nor were there differences in superoxide production (*n* = 8). **(H)** Activity (AUC) of purified monoamine oxidase (MAO) in presence of preferential substrate, tyramine (Tyr), is inhibited by monoamine oxidase inhibitor (MAOI). Creatine (Cr) and guanidinoacetate (GA) are equally good substrates for MAO, but only in the absence of primary amines (*n* = 30/group). **(I)** mRNA expression of PGC-1α and its upstream and downstream regulated genes by real-time quantitative PCR in LV from GAMT^–/–^ and WT mice >1 year age (*n* = 4/group). Fold change normalized to control concentration (WT levels = 1) with propagated errors (SEM). **(J)** Uncoupling protein 3 (UCP3) expression and representative western blot (*n* = 4/group 2F/2M). ^∗^Denotes *P* < 0.05, ^∗∗^*P* < 0.01, ^∗∗∗^*P* < 0.001 versus WT by unpaired *t*-test. Pearson’s correlation coefficient was used to assess the relationship between the variables. Mouse mean age 84 weeks **(C–J)**. All values are mean ± SEM.

Short-term myocardial creatine depletion is known to impact mitochondrial organization in terms of proliferation, function (Wiesner et al., 1999; Chaturvedi et al., 2010) and shortening of diffusion distances between mitochondria and myofilaments (Kaasik et al., 2001). Thus, electron microscopy was used in 1 year old WT and GAMT^–/–^ hearts (*n* = 3/group) to determine whether similar changes occur with prolonged creatine deficiency (Figures 3A,B). Diffusion distance (distance between the sarcomere M-line and the nearest mitochondrial outer membrane) was not significantly different between WT (0.54 ± 0.11 μm) and GAMT^–/–^ (0.62 ± 0.23 μm). However, the percentage of cell volume occupied by mitochondria in GAMT^–/–^ mice was lower (Figure 3C) and was corroborated by lower mitochondrial DNA copy number (Figure 3D; *P* < 0.004), lower activity of mitochondrial CK (Figure 2C) and by an inverse correlation between citrate synthase activity and age in GAMT^–/–^ mice (Figure 3E). Creatine is reported to have direct antioxidant activity (Lawler et al., 2002), whereas GA may generate free radicals (Mori et al., 1996), therefore, GAMT^–/–^ may be more susceptible to oxidative stress. However, no differences were observed in total protein carbonylation or in superoxide production in homogenates from >1 year old hearts (Figures 3F,G). Monoamine oxidases (MAO) are a major source of reactive oxygen species (ROS), generating H_2_O_2_ via deamination of intracellular amines (Di Lisa et al., 2009). It is unknown whether creatine or GA are potential substrates that could act to alter H_2_O_2_ production. However, this is an unlikely mechanism as both acted as equally effective substrates for MAO A in a cell-free assay and did not affect the preferential utilization of primary amines, e.g., tyramine (Figure 3H).

**FIGURE 4:**
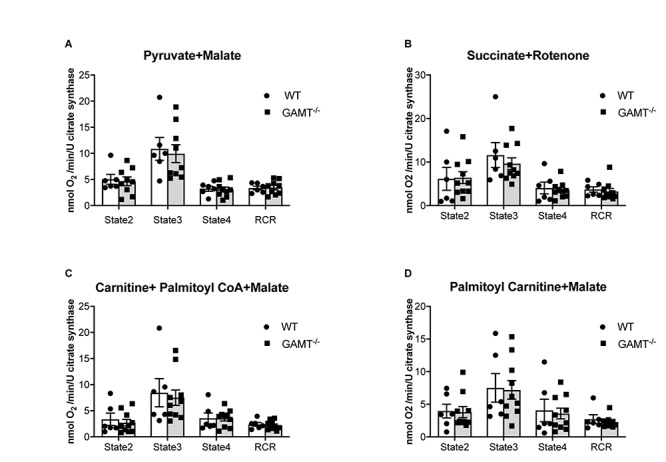
Mitochondrial oxygen consumption. Data from aging GAMT^–/–^ and WT permeabilized LV fibers were normalized to citrate synthase activity to correct for the reduced mitochondrial volume density observed in aging GAMT^–/–^ hearts. The metabolic substrates chosen for mitochondrial respiration were physiological in the presence of a functional Krebs’ cycle intermediate (malate) allowing mitochondrial OXPHOS to proceed normally, providing reduced intermediates (NADH + H^+^ and FADH_2_) individually to complexes I (pyruvate) **(A)** and II (succinate) **(B)**, or combined complex I + II (fatty acid metabolism analogs palmitoyl-carnitine; palmitoyl CoA + carnitine) **(C,D)** of the respiratory chain allowing the thermodynamic cascade through the Q-cycle and complex III to complex IV and O_2_. State 2 is basal unstimulated respiration, State 3 is maximal ADP-stimulated respiration, State 4 is oligomycin uncoupled respiration (ATP synthase activity inhibited), RCR is respiratory control ratio (State3/State4) (WT *n* = 6 3F/3M GAMT^–/–^ *n* = 9 4F/5M). Mouse mean age 72 weeks. All values are mean ± SEM.

PGC1-α is thought to be a master regulator of mitochondrial biogenesis (Finck and Kelly, 2007). In GAMT^–/–^ mice >1 year age, PGC1-α expression was paradoxically up-regulated (2.5-fold), but without accompanying changes in up-stream genes (Sirt1) or down-stream genes involved in mitochondrial biogenesis (Nrf1, Nrf2, PGC1-β, Tfam) (Figure 3I). There was a non-significant trend for increased expression of RIP140 corepressor of nuclear receptors which could act as a brake on PCG1α transcriptional signaling (*P* < 0.056) (Figure 3I). There were no differences in expression for genes involved in regulating metabolic substrate uptake and utilization (GLUT4, GLUT1, CPT1β, PPARα, PDK4), mitochondrial fission and fusion (Drp1, Fis1, Mfn1, Mfn2, Opa1, Mff), or mitophagy (Parkin and Pink1) (Figure 3I). The expression pattern for young mice is shown in Supplementary Figure 2 and suggests that aberrant PGC1-α signaling develops with age in GAMT^–/–^ hearts. Protein expression of uncoupling protein 3 was up-regulated in aging GAMT^–/–^ hearts, which could reflect mitochondrial proton leak (Figure 3J). However, when mitochondrial oxygen consumption was measured in isolated cardiac-fibers using a range of metabolic substrates, the respiratory control ratios (RCR) were broadly normal (Figure 4).

Mitochondrial respiration in the aging GAMT^–/–^ mice was indistinguishable from WT for the whole range of substrates examined when O_2_ consumption was normalized to citrate synthase activity to control for differences in total mitochondrial volume (Figure 4) or dry fiber weight (Supplementary Figure 3). This includes the ability to utilize fatty acids via both CPT1 dependant and independent fatty acid metabolism intermediates (Figures 4C,D).

### Phenotype Rescue by Creatine Supplementation

**FIGURE 5:**
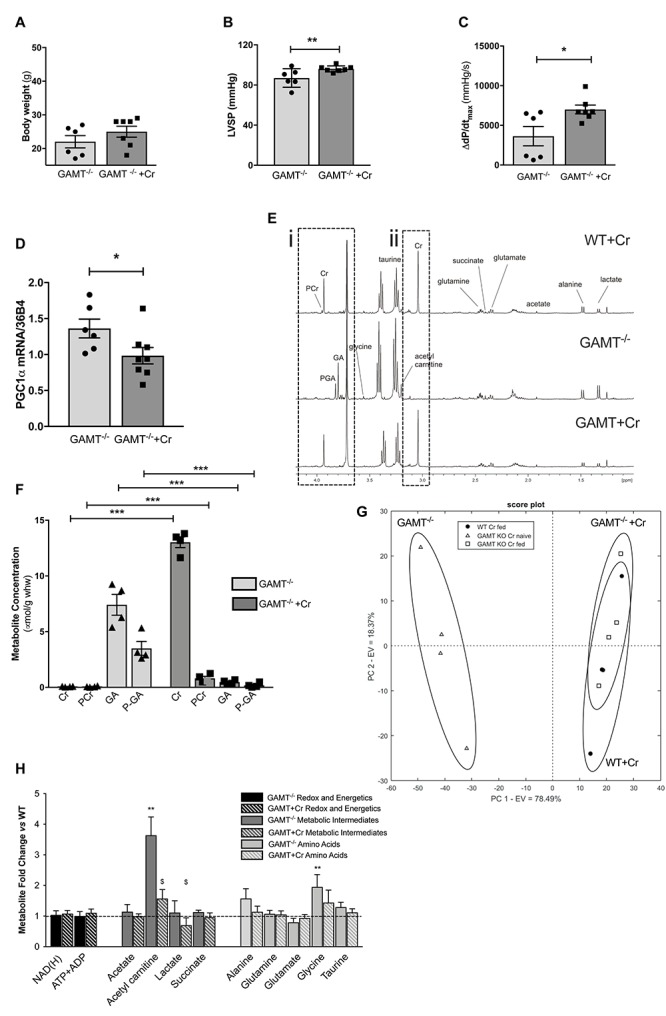
Dietary creatine supplementation for 10 days normalizes the functional and metabolic phenotype of creatine-naïve GAMT^–/–^ hearts Impact of dietary creatine on **(A)** Body weight; **(B)** LV systolic pressure, LVSP; **(C)** contractile reserve, i.e., increase in d*P*/d*t*_*max*_ with maximal β-adrenergic stimulation. Dietary creatine supplementation normalizes PGC1-α gene expression **(D)**, concomitant with a return to normal tissue concentrations for guanidinoacetate (GA), phosphoguanidinoacteate (PGA) **(Ei,F)**, creatine and PCr **(Eii,F)**. Unsupervised principal component analysis of ^1^H NMR derived metabolite concentrations show good separation between GAMT^–/–^ and WT and GAMT^–/–^ Cr-fed groups **(G)**. Metabolite concentrations expressed as fold change compared to WT levels in GAMT^–/–^ and GAMT^–/–^ creatine supplemented hearts **(H)**. (WT *n* = 4 2F/2M GAMT^–/–^ *n* = 6 3F/3M GAMT^–/–^ + Cr *n* = 7 3F/4M). ^∗^*P* < 0.05, ^∗∗^*P* < 0.01, ^∗∗∗^*P* < 0.001 GAMT^–/–^ vs. WT, ^$^GAMT^–/–^ vs. GAMT^–/–^ + creatine by one way ANOVA. Mouse age 60 weeks. All values are mean ± SEM.

A key question is whether *in vivo* functional impairment in GAMT^–/–^ hearts is a consequence of chronic creatine deficiency or due to toxicity from long term GA accumulation? Aging GAMT^–/–^ mice were given normal chow or chow supplemented with creatine monohydrate (0.75% w/w) for 10 days. A small cohort (*n* = 4) of WT mice were also fed creatine to provide baseline reference values, but were not used in the comparison since we had insufficient aged mice to provide statistical power. Replenishing creatine in GAMT^–/–^ mice did not affect body weight (Figure 5A), but improved hemodynamic parameters, correcting the deficits in LV systolic pressure (Figure 5B) and contractile reserve (Figure 5C) (Supplementary Table 2). Furthermore, creatine replenishment reverted myocardial PGC1-α gene expression to WT levels (Figure 5D). ^1^H-NMR spectroscopy in LV homogenates (Figure 5E) confirmed that creatine supplementation completely restored myocardial total creatine levels in GAMT^–/–^ mice (Figure 5F). However, tissue concentrations of GA and P-GA were also unexpectedly normalized. Unsupervised principal component analysis (PCA) of all detected metabolites observed good separation between the GAMT Cr-naïve group (GAMT^–/–^) and the WT and GAMT^–/–^ Cr-fed groups (Figures 5G,H), while the Cr-fed groups were indistinguishable. Therefore, although dietary creatine rescued the hemodynamic and PGC1-α phenotypes, we were unable to disambiguate between creatine-deficiency and GA-toxicity as the causative factor.

### Theoretical Consideration of Myocardial ATP Utilization in GAMT^–/–^ Mice

We have shown that aging GAMT^–/–^ hearts have hemodynamic impairment, but without evidence of adverse LV remodeling or congestive heart failure. Since the GAMT^–/–^ hearts are *de facto* performing less external work, it is useful to ask how this impacts on cardiac energy requirements? This is not straightforward since we have not measured oxygen consumption and the absence of creatine precludes the calculation of free [ADP] from the CK equilibrium reaction and hence ATP flux and ΔG_ATP_ are unknown. Instead, we have used our current and historical datasets to calculate external work (minute work), then taken literature values for ATP production rate and ΔG_ATP_ in order to calculate the percentage of energy production being used to perform external work (akin to cardiac efficiency and providing similar values (Westerhof, 2000; Table 2). These assumptions are unlikely to be valid for GAMT^–/–^ and we have therefore calculated the energy required to maintain WT “efficiency” and hence the relative energy saved by performing less external work. This suggests that at 18 months of age the energy available from ATP can be 33% lower in KO and they will still be able to maintain their current systolic function. This argues for reduction in external work as an adaptation to chronically impaired energy production and/or utilization.

**TABLE 2:** Theoretical consideration of cardiac work and energy requirements with age.

	WT			GAMT^–/–^		
	4–6 months	8–10 months	12–18 months	4–6 months	8–10 months	12–18 months
**Measured parameters**						
Stroke volume (μL)	34	38	38	32	32	34
LV developed pressure (mmHg)	94	98	93	86	79	74
Heart rate (BPM)	523	521	487	490	479	388
LV weight (mg)	85	97	106	69	77	88
**LV work**						
Stroke work (mmHg μL)	3187	3730	3540	2795	2535	2507
Minute work (mmHg ml/min)	1663	1943	1731	1370	1221	969
Minute work (J/min)^∗^	0.222	0.259	0.231	0.183	0.163	0.129
**Energy production**						
ATP production rate for normal murine heart = 3.16 μMol/g/s^∗∗^						
ATP production per LV (μM/s)	0.269	0.305	0.335	0.216	0.243	0.279
ΔG for ATP hydrolysis = 57 kJ/mol^∗∗∗^						
Energy produced (J/min)	0.920	1.045	1.147	0.740	0.830	0.953
Minute work/Energy produced (%)	24.1	24.8	20.1	24.7	19.6	13.6
Energy required to maintain WT ratio (J/min)	0.920	1.045	1.147	0.758	0.656	0.642
Energy saved by reduced Work (%)	n/a	n/a	n/a	−2	21	33

*Stroke work was approximated by multiplying LV developed pressure by stroke volume. ^∗^Conversion factor 1 mmHg ml = 0.0133322 cJ (Schramm, 2010); SV measurements from Schneider et al. (2008). LVDP, HR, LV weight from Ten Hove et al. (2005b); Lygate et al. (2013) and this manuscript. ^∗∗^ΔG value from Lygate et al. (2012). ^∗∗∗^ATP production rate from Gupta et al. (2011) (WT *n* = 12, GAMT^–/–^*n* = 15/age group).*

## Discussion

This is the first study to identify long-term consequences of prolonged creatine deficiency and GA accumulation in the hearts of GAMT^–/–^ mice. We observed a progressive decline in *in vivo* hemodynamic parameters that was temporally related to reduced mitochondrial cell density, while mitochondrial function was preserved. Myocardial *PGC-1*α mRNA levels were elevated, but downstream effector genes were not, suggesting dysfunction in the signaling pathway for mitochondrial biogenesis. Previous changes in phosphotransfer pathways observed in young GAMT^–/–^ mice were no longer apparent in aging animals, where instead, we observed an increase in maximal AK activity, suggesting that compensatory adaptations are modified in response to aging. Reintroduction of dietary creatine rescued the *in vivo* functional deficit and normalized PGC-1α mRNA levels. However, it was not possible to infer causation for creatine deficiency since GA levels were simultaneously normalized, which could also contribute to the phenotypic rescue.

### Is the *in vivo* Functional Decline Pathological or Adaptive?

By 18 months of age GAMT^–/–^ mice exhibit a hemodynamic profile, which consists of lower LV end-systolic pressure, heart rate, and rates of contractility and relaxation compared to age-matched controls. This is in contrast to 6 month old GAMT^–/–^ where only LVSP is reduced, suggesting a progressive decline with age (Ten Hove et al., 2005b). It is therefore tempting to conclude that these mice develop cardiomyopathy, however, the associated pathological changes are absent. For example, LV end-diastolic pressures were normal in aging GAMT^–/–^, as were RV and lung weights indicating the absence of pulmonary congestion. Nor was there evidence for progressive LV remodeling, since indices of LV hypertrophy have not changed with age and we have previously shown using cine-MRI that LV chamber diameters are not altered at 1 year (Schneider et al., 2008). Nevertheless, due to difference in body weight, GAMT^–/–^ mice do have elevated LV/body weight ratio throughout adulthood. Thus, in GAMT^–/–^ there is relatively more myocardial tissue available to meet the metabolic demands of the body and this may represent an additional compensatory mechanism to long-term creatine deficiency. The observation that GAMT^–/–^ did not increase mortality (Supplementary Figure 1) and the rapid reversal of hemodynamic phenotype with dietary creatine is inconsistent with chronic pathological maladaptation. Instead, we propose that reduced stroke work in GAMT^–/–^ mice represents a compensatory adaptation to reduce energy requirements, and calculate that the GAMT^–/–^ heart requires 33% less energy from ATP to perform their external work (Table 2).

Why then only in aging mice? Mice are considered middle-aged by 1 year and by 18 months are equivalent to 62 human years, having past the reproductive phase and on the cusp of senescence (Dutta and Sengupta, 2016). It is likely that potential adaptations observed in young GAMT^–/–^ mice, e.g., increased glycolytic enzymes (Ten Hove et al., 2005b) are subject to age-related decline and are therefore not sustainable in the long term. For example, myocardial CK activity in rats has been shown to fall by 25% at 16–24 months (Chesky et al., 1980). This may explain why some enzyme activities were normalized in the aging GAMT^–/–^ heart, while others became elevated, since the compensatory response must adapt to the aging heart. In this context, saving energy via reduction in cardiac work may represent an effective compensatory strategy, particularly in sedentary, post-reproductive lab mice, fed *ad libitum* and housed in stable social groups, where it is essentially consequence-free. Similar hemodynamic alterations have also been described in rats depleted of creatine by β-GPA feeding. These had lower LVSP, d*P*/d*t*_*max*_, d*P*/d*t*_*min*_, and heart rate at rest and during exercise, yet exercise performance was unaffected, suggesting positive adaptations akin to those seen with endurance training (Adams et al., 1995). These include AMPK activation in sinoatrial cells, which has been shown to lower resting heart rate in order to reduce cardiac energy demand (Yavari et al., 2017). However, our phenotype may have been even more robust if we had studied mice older than 18 months.

### Chronic Creatine Deficiency and Mitochondrial Pathology

Creatine is functionally coupled to mitochondrial respiration, while in the hearts of CK knockout mice, the mitochondria are repositioned to minimize ATP diffusion distances (Kaasik et al., 2001). Depletion of intracellular creatine by β-GPA in cultured rat cardiomyocytes results in mitochondria that are morphologically larger and rounder with “paracrystalline inclusions” within the christae (Eppenberger-Eberhardt et al., 1991). Similar mitochondrial morphology was described in skeletal muscle after 6 months of β-GPA feeding in rats (De Tata et al., 1993). However, no such changes in mitochondrial organization or structure were observed in hearts from GAMT^–/–^ mice at 45 weeks of age (Branovets et al., 2013), nor in the present study. Instead, electron microscopy showed lower mitochondrial volume density in knockout heart, which was confirmed by reduced mitochondrial DNA and citrate synthase activity.

An important question is whether this is pathological or adaptive? We did not observe differences in expression of genes involved in mitophagy or in mitochondrial fusion and fission. However, there are several hypothetical mechanisms for increased oxidative damage in GAMT^–/–^ mice, for example, direct generation of hydroxyl radicals by GA has been described (Mori et al., 1996), as has a direct antioxidant effect of creatine, at least *in vitro* (Lawler et al., 2002; Aksentijevic et al., 2014b). Creatine and/or GA have the potential to modify the two largest sources of cellular ROS, i.e., superoxide anions (O_2_^–^) as a by-product of electron-transport chain activity (Cadenas and Davies, 2000), and the production of hydrogen peroxide (H_2_O_2_) by amine oxidases (Cadenas and Davies, 2000; Agostinelli et al., 2004).

However, we were unable to detect any difference between WT and GAMT^–/–^ hearts in terms of myocardial O_2_^–^ generation, protein carbonylation as a marker for cumulative oxidative damage, or in mitochondrial respiration under baseline and stimulated conditions. Although UCP3 expression was elevated in GAMT^–/–^ hearts, this was not reflected in altered RCR. This is in contrast to young GAMT^–/–^ where RCR was impaired with no change in UCP3 expression. This suggests the presence of compensatory adaptations that develop with age, but unfortunately does not provide further mechanistic insight. Finally, we demonstrated for the first time that both creatine and GA are substrates for monoamine oxidase, however, both are equally good substrates and they do not compete significantly with primary amines which are the substrates of choice. Overall our data does not support oxidative stress as a mediator of cardiac mitochondrial pathology in GAMT^–/–^ hearts. Low mitochondrial cell density could also arise from impaired biogenesis. We therefore measured gene expression of the key regulator, *PGC1-*α, which was paradoxically up-regulated 2.5-fold in GAMT KO mice >1 year, but not in younger mice. However, none of the downstream PGC1-α effector genes were altered, including *TFAM*, which is involved in mtDNA gene replication (Finck and Kelly, 2007).

In contrast, *RIP140*, which counteracts the effects of *PGC1-*α, showed a trend to be higher in knockout hearts. This suggests that aging GAMT^–/–^ hearts have a dysfunction in PGC1-α signaling, i.e., the signal for mitochondrial-biogenesis is switched on, but is not propagated further.

### Comparison With Other Models of Creatine Depletion

Our findings are in contrast to previous studies that have depleted creatine by feeding β-GPA. For example, rats treated with β-GPA for 6 weeks had increased mitochondrial cell density in the heart, associated with increased expression of mitochondrial proteins and *TFAM* (Wiesner et al., 1999). In a similar protocol, β-GPA also stimulated mitochondrial biogenesis pathways in skeletal muscle via activation of AMPK, which lies upstream of *PGC1-*α, although the effect was modulated in different muscle types by the expression of *RIP140* (Williams et al., 2009). It is notable that an increase in mitochondrial biogenesis has also been described in aging skeletal muscle, probably in response to accumulation of mitochondrial DNA mutations and dysfunctional proteins (Herbst et al., 2013). It is suggested that this promotes a vicious cycle, since biogenesis results in more mutations, thereby promoting dysfunction and further biogenesis. If the same thing happens in heart, then the GAMT^–/–^ would be uncoupled from this cycle.

It seems likely that the consequences of creatine depletion depend on whether ATP levels fall, for which the activation of AMPK represents a biomarker. For example, β-GPA feeding increased *PGC1-*α expression and mitochondrial biogenesis in skeletal muscle of wild-type mice, but not in mutant mice expressing non-functional AMPK (Zong et al., 2002). Notably, myocardial ATP levels are preserved in GAMT^–/–^ hearts and AMPK is not activated (Lygate et al., 2013). This is also true for creatine-free AGAT^–/–^ mice, which exhibit contractile dysfunction, but as a consequence of homoarginine deficiency rather than creatine (Faller et al., 2018). In contrast, ATP is depleted in AGAT^–/–^ skeletal muscle with activation of AMPK, resulting in a severe skeletal muscle pathology that is completely rescued by dietary creatine (Nabuurs et al., 2013; Faller et al., 2018). A fall in myocardial ATP levels has also been described in rat hearts after 12 weeks β-GPA, and although this was associated with adaptations likely to improve energy efficiency, they nevertheless developed cardiac hypertrophy (Mekhfi et al., 1990), which isn’t observed in the genetic models.

Therefore, the preservation of myocardial ATP levels in GAMT^–/–^ and AGAT^–/–^ hearts is likely to reflect chronic compensatory adaptations, when compared to the β-GPA feeding studies, which are relatively short-term, and result in gradual and only partial creatine depletion. However, we cannot rule out a role for differences in the potential toxicity of GA versus β-GPA accumulation.

### Creatine Rescue of the Aging GAMT^–/–^ Phenotype

GAMT^–/–^ mice, in common with human GAMT deficiency, have a unique metabolic fingerprint of zero creatine accompanied by chronic GA/P-GA accumulation. The first human case was described in 1994 (Stockler et al., 1994) with ∼80 cases recorded to date (Akiyama et al., 2014; Stockler-Ipsiroglu et al., 2014). It manifests at an early age in the form of developmental delay and neurological symptoms, to an extent that the effect on the heart has not been studied. Notably, human GAMT deficiency is associated with severe epilepsy that does not improve with creatine-supplementation, but does respond to GA lowering strategies (Schulze, 2003). This has been confirmed in GAMT^–/–^ mice, which have a reduced seizure threshold normalized by GA reduction (Schulze et al., 2016). Direct injection of GAA into brain or exposure of brain samples has also demonstrated acute toxicity independent of creatine levels, e.g., increasing acetylcholinesterase activity to modify behavior (Zugno et al., 2008a); reducing antioxidant capacity (Zugno et al., 2008b); inhibition of the Na^+^, K^+^-ATPase (Zugno et al., 2003). Our previous work has shown P-GA can be utilized under the extreme conditions of prolonged ischemia, but is regenerated very slowly (Ten Hove et al., 2005b). We measured CK flux using ^31^P-NMR and showed that while P-GA does accumulate in GAMT^–/–^ heart (presumably via participation in the CK reaction), we were unable to detect ATP generation from P-GA suggesting that this is not a rapid or efficient mechanism under non-ischemic conditions (Lygate et al., 2013).

We therefore sought to determine whether the changes we observed in GAMT^–/–^ hearts were driven by chronic creatine deficiency or the toxic effects of GA accumulation. By re-introducing dietary creatine we expected (and indeed observed) a rapid restoration of myocardial creatine levels, since GAMT^–/–^ hearts are known to upregulate the creatine transporter (CrT) (Ten Hove et al., 2008). Over the same timeframe, our expectation was for GA levels to barely change, since we assumed that cellular efflux would be slow and passive as it is for creatine (Wyss and Kaddurah-Daouk, 2000).

However, GA levels fell to undetectable levels, meaning that, although creatine supplementation normalized the hemodynamic profile and PGC1-α gene expression, we were unable to infer causation. We found this outcome highly surprising as we fully expected that within this time-scale creatine levels would go up, while GA levels would barely change, which would have allowed us to assign causation.

With hindsight, we could have attempted phenotypic rescue using dietary ornithine, a competitive inhibitor of AGAT, which has been shown to lower plasma GA levels by 44% over 18 days in GAMT^–/–^ (Schulze et al., 2016). Whether this reduction would be sufficient is moot, but it may feasibly have provided additional insight into GA toxicity.

We have previously estimated creatine efflux from the heart to be 2.7% per day via non-enzymatic degradation (Faller et al., 2018), but GA efflux was evidently much more rapid. While there may be some spontaneous hydrolysis of GA to urea and glycine (which could explain the trend toward elevated glycine in GAMT^–/–^ hearts), there is no known active mechanism for GA degradation as occurs in microorganisms (via guanidinoacetate amidinohydrolase) (Shirokane et al., 1987). GA is a known substrate for the CrT, but it is unlikely to work in reverse under normal physiological conditions (Ten Hove et al., 2005a). However, GA also appears to be a substrate for the taurine transporter, at least at the blood-brain barrier (Tachikawa et al., 2009), so one possibility is the taurine transporter working in reverse to balance the osmotic effect of creatine entry (Han et al., 2006). Creatine also exerts end-product inhibition on AGAT (Derave et al., 2004), which means GA will not be replenished during exogenous creatine supplementation. Finally, in the absence of creatine, a significant proportion of GA is phosphorylated and thereby membrane impermeable, but creatine is the preferred substrate for CK, so as creatine enters there will be progressively less GA in the phosphorylated form.

## Conclusion

Creatine-deficient GAMT^–/–^ hearts exhibit a blunted hemodynamic profile with age that is associated with reduced mitochondrial cell volume and aberrant PGC1-α signaling. However, it was not possible to differentiate between creatine-deficiency or guanidinoacetate toxicity as causative. Nevertheless, mortality and mitochondrial respiration were normal, and the absence of other markers of cardiomyopathy suggest these changes are ultimately benign. This supports our previous data in genetic mouse models that creatine-deficiency *per se* does not directly cause heart failure, but suggests that compensatory strategies may adapt to the aging heart. Since the lower cardiac stroke work observed in aging GAMT^–/–^ is energy saving, this may, in fact, represent an acceptable compensatory trade-off in sedentary mice that are past reproductive age.

## Materials and Methods

### Animal Husbandry

Male and female GAMT knockout mice on a pure C57BL/6J OlaHsd genetic background were genotyped as previously described (Schmidt et al., 2004). Breeding was by heterozygous mating to provide littermates as wild type (WT) controls. Post-weaning, GAMT^–/–^ and WT mice were housed separately to prevent creatine absorption via coprophagia. Mice were fed standard chow (naturally creatine-free), water *ad libitum* and kept in specific pathogen-free conditions, with 12h light–dark cycle at 20–22°C.

### Compliance With Ethical Standards

This investigation was approved by the Committee for Animal Care and Ethical Review at the University of Oxford and conforms to the UK Animals (Scientific Procedures) Act, 1986 (Home Office project licence 30/3314), incorporating Directive 2010/63/EU of the European Parliament and conforms to European Convention for the Protection of Vertebrate Animals used for Experimental and other Scientific Purposes’ (Council of Europe No 123, Strasbourg, 1985).

### *In vivo* Cardiac Assessment

Mice aged 72 weeks were anesthetized with 4% isoflurane in medical O_2_ and maintained on a nose-cone at 1–1.5% for left ventricular (LV) hemodynamic measurements. LV was cannulated via the right carotid artery using a 1.4F Millar micro-tip cannula (SPR-839, Millar Instruments, Houston, TX, United States). Measurements were obtained after 15 min equilibration via a Powerlab 4SP data acquisition system (AD Instruments, United Kingdom) (Aksentijevic et al., 2010). For LV tissue collection, mice were euthanized by intra-peritoneal injection of pentobarbitone 140 mg/kg. At the end of the hemodynamic examination the hearts were removed under deep isoflurane anesthesia.

### Electron Microscopy

Hearts were excised from male mice aged 50 weeks (full details in Supplementary Material) and 19–31 fields-of-view from LV (*n* = 3 WT and GAMT^–/–^) were analyzed using ImageJ software by a single operator blinded to genotype. Volume fractions were calculated using a standard grid method and expressed as percentage of total cell volume (Weibel et al., 1966).

### Mitochondrial Function Experiments

Saponin–permeabilized cardiac fibers isolated from the LV endocardium (age 72 weeks) were used to measure respiration of the total mitochondrial population *in situ* using a Clark-type oxygen electrode (Strathkelvin Instruments, United Kingdom) (Lygate et al., 2013) (full details in the Supplementary Material).

### Metabolic Profile Analysis

Snap frozen heart tissue (mean age 84 weeks) was used for metabolic profiling using previously published protocols: high resolution ^1^H NMR spectroscopy (Chung et al., 2017), creatine kinase (Aksentijevic et al., 2014b) adenylate kinase (AK_*total*_, isoforms AK_1_ and AK_*remnant*_) and glycolytic enzyme activities [glyceraldehyde-3-phosphate dehydrogenase (GAPDH), 3-phosphoglycerate kinase (PGK), and pyruvate kinase (PK)] assessments (Aksentijevic et al., 2010), pyruvate dehydrogenase (PDH) assay, myocardial glycogen and triacylglycerol (TAG) content assessment (Aksentijevic et al., 2014a), HPLC analysis of the total adenine nucleotide pool (ATP + ADP + AMP), creatine and phosphocreatine (PCr) (Aksentijevic et al., 2010). Method for the spectrophotometric assessment of maximal F_1_-ATPase hydrolytic activity in isolated mitochondria was developed for the purpose of this study (Supplementary Material). Plasma was collected from terminally anesthetized non-fasting mice and metabolic profile analyzed as described in Supplementary Material.

### *In vitro* Activity of Monoamine Oxidase A

The potential involvement of creatine and guanidinoacetate in H_2_O_2_ production *via* MAO-A (Sigma Aldrich Poole, United Kingdom) was evaluated using Amplex^®^ red reagent hydrogen peroxide/peroxidase fluorescence assay kit (Invitrogen, Paisley, United Kingdom). It was previously shown that the use of MAO-A substrate tyramine allows evaluation of H_2_O_2_ production in substrate concentration dependant manner (Mazzio and Soliman, 2004) and this effect is irreversibly inhibited by phenelzine (Riederer et al., 2004; Youdim et al., 2006; Di Lisa et al., 2009).

### Gene and Protein Expression Analysis

Total protein was extracted from snap-frozen LV tissue and analyzed by immunoblotting as described previously (Lygate et al., 2013). For carbonylation, 20 μg of protein were derivatized using a protocol adapted from Divald et al. (2010), as previously reported (Aksentijevic et al., 2014b) (Supplementary Material). Messenger RNA expression levels were tested by qRT-PCR for 3 panels of gene groups, namely mitochondrial biogenesis, metabolic substrate utilization and fusion/fission genes as described in Supplementary Table 3.

### Dietary Creatine Supplementation Study

Standard laboratory chow (Teklad global 16% rodent diet) was powdered and re-formed into “cookies” with, or without, addition of creatine monohydrate 0.75% w/w and using a small quantity of water to bind. A cohort of 60 week old GAMT^–/–^ and matching WT controls were first fed creatine-free cookies for 2–3 days to accustom mice to the change in food appearance and texture, then maintained on creatine-free or switched to creatine-supplemented cookies for a further 10 days. Creatine dose was based on Kan et al. (2007) assuming a 20 g mouse eating 4 g chow per day. It is also the dose that was previously shown to correct LV creatine levels in GAMT^–/–^ mice (Lygate et al., 2013). A small number of WT mice were included to determine any effect of creatine-supplementation unrelated to correcting creatine deficiency. At the end of the feeding protocol *in vivo* LV hemodynamic measurements were performed under isoflurane anesthesia at baseline and with maximal β-adrenergic stimulation using dobutamine 16 ng/g body weight/min as described previously (Lygate et al., 2013).

### Data Analysis

All data was analyzed blind to genotype. Comparison between two groups was by Student’s *t*-test (Gaussian data distribution), Welch’s *t*-test (non-equal standard deviation distribution) and between 3 groups by one-way analysis of variance (ANOVA) using Bonferroni’s correction for multiple comparisons. Pearson’s correlation coefficient was used to assess the relationship between the variables. Data are presented as mean ± SEM. Statistical analysis was carried out using GraphPad Prism software, v.8.0. Differences were considered significant when *P* < 0.05.

## Data Availability Statement

The datasets generated for this study are available on request to the corresponding author.

## Ethics Statement

The animal study was reviewed and approved by the Committee for Animal Care and Ethical Review at the University of Oxford and conforms to the UK Animals (Scientific Procedures) Act, 1986, incorporating Directive 2010/63/EU of the European Parliament. Home Office project licence 30/3314.

## Author Contributions

DA, CL, SZ, and SN designed the research. DA, SZ, TE, DM, JW, JS, and CL performed the research and analyzed the data. DA and CL involved in data interpretation and manuscript writing.

## Conflict of Interest

The authors declare that the research was conducted in the absence of any commercial or financial relationships that could be construed as a potential conflict of interest.
