# Supplementary material for: Age-Dependent Decline in Cardiac Function in Guanidinoacetate-N-Methyltransferase Knockout Mice
Source: Front Physiol. 2020 Jan 21;10:1535. doi: 10.3389/fphys.2019.01535 (PMC6985570; doi:10.3389/fphys.2019.01535)
Supplement: Supplementary file 1 [file Data_Sheet_1.docx]

**Supplementary Material**

**Supplementary Results**

**Supplementary Table 1. Six month old GAMT^-/-^ myocardial biochemistry**

*P<0.05 vs WT by unpaired t-test. (n=4-12)

|  | **WT** | **GAMT^-/-^** |
| --- | --- | --- |
|  | (n=4-12) | (n =4-12) |
| ***Biochemical Profile*** | | |
| CK activity (U/mg) | 8.3±0.4 | 9.1±0.6 |
| TAN pool (nmol/mg protein) | 32.9±3.1 | 29.9±2.9 |
| PDHa/PDHt (μmol/min/g whw) | 3.5±0.6 | 3.8±0.7 |
| F_1_-ATP synthase (μmol/min/mg) | 2.2±0.19 | 4.2±0.4* |
| TAG (mM/g whw) | 0.07±0.004 | 0.04 ± 0.003* |
| Glycogen (glycolsyl units/g whw) | 2.6±0.4 | 4.7±0.4* |

Whw- wet heart weight CK- creatine kinase, TAN pool (total adenine nucleotide pool ADP+ATP+AMP), PDH-pyruvate dehydrogenase, PDHa- pyruvate dehydrogenase active PDHt- pyruvate dehydrogenase total, TAG-triacylglycerol

**Supplementary Table 2. Haemodynamic parameters in GAMT^-/-^ >1 year after Cr rescue at baseline and post-dobutamine stress**

|  | **GAMT^-/-^** | **GAMT^-/-^ +Creatine** |
| --- | --- | --- |
|  | (n = 3M/3F ) | (n = 4M/3F) |
| Age (weeks) | 61±2 | 62±2 |
| Body weight (g) | 22±2 | 25±2 |
| *Baseline* |  |  |
| End systolic pressure (mmHg) | 84.7±1.1 | 92.2±1.7** |
| End diastolic pressure (mmHg) | 4.4±0.8 | 4.9±1.1 |
| dP/dtmax (mmHg/s) | 6418±440 | 7668±797 |
| dP/dtmin (mmHg/s) | -6433±734 | -8814±1102 |
| Heart rate (bpm) | 437±30 | 477±43 |
| Tau (ms) | 7.7±0.8 | 6.8±1.3 |
| *Dobutamine* |  |  |
| End systolic pressure (mmHg) | 88.9±3.7 | 96.0±1.1 |
| End diastolic pressure (mmHg) | 3.3±0.34 | 3.7±0.29 |
| dP/dtmax (mmHg/s) | 10052±1310 | 14663±728 *** |
| dP/dtmin (mmHg/s) | -7931±815 | -10417±819 |
| Heart rate (bpm) | 536±39 | 614±26 |
| Tau (ms) | 5.9±0.8 | 4.6±0.3 |
| ΔdP/dtmax (mmHg/s) | 3634±1218 | 6996±555 ** |

Data are mean ± S.E.M. GAMT^-/-^ vs GAMT ^-/-^+Cr ***P<0.008 *P<0.05 **P<0.02 by one way ANOVA

**Supplementary Figure 1.** Chronic creatine deficiency is not associated with increased mortality up to 500 days of age**.** WT n=120 (females n=50, males n=70) GAMT^-/-^ n=122 (females n=53, males=69). Data presented is a combination of male and female mice. Mice have been censored at time of terminal experiments (shown as up-tick).

**Supplementary Figure 2. Myocardial gene expression in 6 month old GAMT^-/-^**

P<0.01 vs WT by unpaired t-test. (n=4/group)

**Supplemental Figure 3. Mitochondrial respiration rates in GAMT^-/-^ >1 year of age**

Respiration rates normalized to gram dry fibre weight. Data are mean ± S.E.M. *P<0.05 vs GAMT^-/-^ by T-test, WT n=6 3F/3M GAMT^-/-^ n=9 4F/5M

**Supplementary Methods**

***Plasma Collection***

Blood was collected from terminally anaesthetized non-fasting mice GAMT KO (n=5-7) and WT mice into EDTA microcapillary tubes (Sarstedt, UK) and centrifuged at 4°C (3000 rpm, 10 min) to obtain plasma. Concentrations of free fatty acids, triglycerides, cholesterol, high-density lipoprotein, low-density lipoprotein, total creatine kinase (CK), lactate, lactate dehydrogenase (LDH), and 3-hydroxybutyric acid (ketone bodies) were measured by the Mouse Biochemistry Laboratory, Addenbrooke’s Hospital, Cambridge University Hospitals NHS Trust.

***Electron Microscopy***

Hearts were excised from male mice aged 50 weeks and retrogradely perfused with heparinised calcium Tyrode solution (containing in mM: NaCl 140.0; KCl 5.4; MgCl_2_ 1.0; HEPES 5.0; Glucose 11.0; CaCl_2_ 1.8), followed by a cardioplegic arrest with Tyrode solution containing 20mM KCl. Hearts were perfused with Karnovsky’s fixative (for 200ml: 4.0g paraformaldehyde, 4.28g sodium cacodylate, 8.0ml 50% glutaraldehyde) and left in fixative overnight at 4°C. The following day, LV was dissected free of other structures and a mid-ventricular portion cut into a <3mm thick ring and subsequently washed in cacodylate buffer. 19-31 fields-of-view from n=3 WT and GAMT^-/-^ mice were analysed using ImageJ software by a single operator blinded to genotype. Volume fractions were calculated using a standard grid method and expressed as percentage of total cell volume (Weibel et al., 1966).

***Mitochondrial Function Experiments***

A piece of tissue was cut from the endocardial free wall of the LV and transferred to a pre-cooled Petri dish with ice cold relaxing and biopsy preservation solution (BIOPS) containing 10mM Ca^2+^-EGTA buffer, 0.1 μM free Ca^2+^, 20 mM imidazole, 20 mM taurine, 50 mM K-MES, 0.5 mM DTT, 6.56 mM MgCl_2_, 5.77 mM ATP, 15 mM phosphocreatine, pH 7.1. Individual fiber bundles were separated with sharp forceps and permeabilized in saponin containing BIOPS (50 mg/ml) for 20 min at 4°C .The presence of saponin perforated sarcolemma while leaving the mitochondria morphologically and functionally intact. Permeabilized fibers were washed in respiration medium containing (in mM): EGTA 0.5, MgCl_2_.6H_2_O 3, k-lactobionate 60, taurine 20, KH_2_PO_4_ 10, HEPES 20, sucrose 110, fatty acid free-bovine serum albumin 1 g/l, pH 7.1. Individual bundles were transferred into respiration chamber containing air-saturated, continuously stirred respiration medium at 37°C.

Mitochondrial respiration was assayed using pyruvate (10mM) with malate (5mM), glutamate (10mM) with malate (5mM), succinate (10 mM) with rotenone (0.5mM), palmitoyl CoA (40µM) with carnitine (5mM) and malate (5mM), and palmitoyl carnitine (40µM) with malate (5mM). Following the measurement of basal respiration (state 2), state 3 (maximal ADP-stimulated) respiration was measured after addition of 2mM ADP. Post-oligomycin (uncoupled state 4) respiration was evaluated following the addition of 1µg/ml oligomycin to inhibit ATP synthase or antimycin A in case of succinate stimulated respiration. Subsequently, fibers were removed, blotted and dried for 24h at 37ºC and dry weight recorded. The Strathkelvin 782 System v 4.4 software was used for data acquisition and analysis. Respiration rates are expressed as nmol O_2_/minute/milligram dry weight of fibers. Respiratory control ratios (RCR) were calculated as a ratio of state 3/state 4 respiration.

***F_1_-ATPase Assay***

Mitochondria were isolated from GAMT^-/-^ and wild type hearts (27 weeks old, n=6) based on the method of Palmer *et al* (Palmer et al., 1977). In brief, LV tissue was minced in ice-cold isolation medium (IM) (Sucrose 0.3M, Na-Hepes 10 mM, EDTA 0.2 mM, pH 7.2) and briefly homogenized (Kinematica Polytron, Phillip Harris Scientific, Lichfield, UK). Trypsin was added (5mg/g wet weight) and the tissue incubated for 10 min at 0˚C. The digestion was halted by adding 1 ml IM containing trypsin inhibitor (5mg/g) (Invitrogen, Paisley, UK). The buffer was decanted, tissue suspended in IM and re-homogenized (20 sec, setting 6). Homogenate was centrifuged at 2500rpm for 10 min, 4˚C (Beckman Coulter J6-MC, Luton, UK), supernatant collected and further centrifuged at 8500 rpm for 10 min, 4˚C (Heraeus Intruments Biofuge Pico, Osterode, Germany). The resultant pellet was washed in low salt F1- ATPase extraction buffer (Hepes 20mM, MgCl_2_ 1mM, EGTA 2mM, pH 7.2) and re-homogenized in the low salt extraction buffer in preparation for the F1- ATPase assay and the protein concentration estimation using Biorad assay (Biorad Laboratories, Munich, Germany). Successful mitochondrial enrichment was demonstrated by an increase in citrate synthase activity. Maximal F_1_-ATPase hydrolytic activity was assayed spectrophotometrically at 340nm, 37˚C in homogenized mitochondrial extract by coupling ATP hydrolysis to NADH oxidation (Rosing et al., 1975;Das and Harris, 1990;Scholz and Balaban, 1994). The assay buffer contained (in mM) sucrose 83, EGTA 2, ATP 2, Tris acetate 33, MgCl_2_ 10, phosphoenolpyruvate 1.5, NADH 0.17, pyruvate kinase 6U and lactate dehydrogenase 12U. The difference in reaction rates with and without added oligomycin (100nM/µg) represents F_1_- ATPase activity. The assay buffer was low in Ca^2+^ and Na^+^ to limit any contribution from ATP-ases other than F_1_- ATPase (Das and Harris, 1990). To verify this assumption the assay was tested in the presence of Na^+^/K^+^ATPase inhibitor oubain octahydrate (10µM) and Ca^2+^ ATPase and Na^+^/K^+^ATPase inhibitor sodium orthovanadate (3µM). Less than 10% of the overall F1- ATPase activity was due to contribution from other ATPases. The measured activity was insensitive to the uncoupler carbonyl cyanide-p-trifluormethoxy-phenylhydrazone (FCCP 2µM), suggesting that no proton motive force was generated by the mitochondrial preparation and the F_1_F_0_ ATP synthase accessible to the assay.

***In-vitro activity of monoamine oxidase A***

The potential involvement of creatine and guanidinoacetate in H_2_O_2_ production *via* MAO-A (Sigma Aldrich Poole, UK) was evaluated using Amplex® red reagent hydrogen peroxide/ peroxidase fluorescence assay kit (Invitrogen, Paisley, UK). It was previously shown that the use of MAO-A substrate tyramine allows evaluation of H_2_O_2_ production in substrate concentration dependant manner (Mazzio and Soliman, 2004) and this effect is irreversibly inhibited by phenelzine (Riederer et al., 2004;Youdim et al., 2006;Di Lisa et al., 2009).

The substrates (tyramine, creatine, guanidinoacetate) and inhibitor were prepared in PBS (pH 7.4) and added to the reaction buffer consisting of 50µM Amplex® red and 0.1 U/ml horseradish peroxidise in PBS pH 7.4. H_2_O_2_ production was initiated by addition of 0.4U/ml MAO-A, measured as fluorescent intensity with the excitation at 544 nm and emission recorded at 595 nm, 37°C, using a POLARstar plate reader (BMG Labtech, Germany). MAO-A activity was first tested by the generation of H_2_O_2_ with increasing tyramine concentration (final concentration of 0.001, 0.003, 0.02, 0.1 and 0.8 mM). 0.1 mM tyramine was chosen as the optimal substrate concentration for the assay based on the steady state kinetics of the reaction. Subsequently, MAO-A induced H_2_O_2_ production was measured in the following treatment groups i) 3.3 mM creatine + 0.1mM tyramine, ii) 0.8mM guanidinoacetate+0.1mM tyramine, iii) 7mM phenelzine+0.1mM tyramine iv) 3.3mM creatine v) 0.8mM guanidinoacetate. MAO-A steady state kinetic parameters were evaluated using a linear regression curve fitting analysis.

***Protein Carbonylation***

Derivatized samples were run on a pre-cast 12% SDS gel (Thermo Scientific) at 100V for 90 minutes in HEPES buffer (Thermo Scientific) and prior to transferring proteins onto a PVDF membrane (GE Healthcare, Amersham, UK). Membranes were blocked in 1% BSA in PBS/Tween-20(0.1%) for 1hr at RT, followed by probing with a primary anti-DNPH antibody at 1:150 (Millipore) for 1 hr at RT. For detection of DNPH epitopes, a secondary anti-mouse IgG, HRP-conjugated antibody was used (Millipore). Immunoblotted proteins were detected by chemiluminescence using the ECL Advance Kit according to manufacturer’s protocol (GE Healthcare). Whole lane intensity was estimated by spot densitometry using FluoChem 8800 (Alpha Innotech Corporation). For protein normalization purposes, carbonylation blots were stripped and re-probed using an anti-α-actinin antibody (Sigma) as previously described (Aksentijevic et al., 2014). DNPH lane intensity/α-actinin was recorded per sample.

***Protein expression***

Total protein was extracted from frozen, ground LV tissue using cOmplete^TM^ protease inhibitors (Roche, UK)] and homogenised in a glass tissue homogeniser as previously described (Aksentijevic et al., 2014). The extract was centrifuged at 13,000rpm for 10 min at 4 ^ο^C and the supernatant was analysed for protein concentration using the BCA protein assay (Pierce, UK). Ten µg were analysed by SDS-PAGE prior to wet protein transfer onto PVDF membrane (GE Health Care, UK) at 200 mA overnight at 4 ^ο^C. Protein expression of UCP3 was determined with anti-UCP3 antibody (Abcam, UK). To confirm equal loading of protein, the membrane was stripped of antibodies (stripping buffer 2% SDS, 62.5 mM TRIS-HCl pH 6.8, 100 mM 2-mercaptoethanol) at 55 ^ο^C for 20 min with constant rotation, followed by immunostaining with mouse anti-mouse α-actinin (Sigma-Aldrich, UK), or anti-VDAC1 (Abcam, UK) as above. For pAMPK/AMPKα detection, protein was extracted from freshly obtained snap-frozen tissue samples in the presence of protease and phosphatase inhibitors (PhosSTOP^TM^; Roche). Immunoblots were first probed with pAMPK antibody (detects phosphorylated epitope at threonine 172) and then re-probed with AMPKα according to manufacturer’s manual (Cell Signaling Technologies) (Faller et al., 2018). For quantification, the ratio pAMPK/AMPKα was calculated. All secondary HRP-labelled antibodies were purchased from Promega. Protein signal was detected and quantitated using ECL advance chemiluminescence kit (GE Health Care, UK) and the FluorChem 8800 imager respectively. For protein carbonylation**,** protein was extracted from snap-frozen heart samples and quantified as described before (ten Hove et al., 2008). 20µg protein per sample were derivatized to 1x 2,4 Dinitro phenyl hydrazine (DNPH; Camlab Chemicals, Cambridge, UK) in 100% trifluoroacetic acid (TFA; Thermo Scientific) as previously described (Divald and Powell, 2006;Divald et al., 2010).

***Real-time quantitative Reverse Transcriptase-Polymerase Chain Reaction***

Total RNA was extracted from snap-frozen heart tissue of GAMT^-/-^ (*n*=4) and WT (n=3) using the RNeasy Fibrous Tissue Kit (Qiagen, UK) as described previously (ten Hove et al., 2008). Drp1 and Fis1 oligonucleotides were previously reported.(Shirendeb et al., 2012) The oligonucleotide sequences are listed in the Supplemental Table 1.

5ng total RNA were used as input in one-step RT and amplification reactions using the Qiagen Quantitect SYBR Green RT-PCR kit (Qiagen, UK) on the Rotor-Gene system (Corbett Research Ltd, Qiagen, UK) (Aksentijevic et al., 2014). The oligonucleotide sequences are listed in the Table 1 data supplement below. Either the sense or the antisense oligonucleotide per pair, were designed to span intron-exon boundaries on the cDNA sequence. For data analysis, the double-standard curve method was employed, in which standard curves spanning five log dilutions of heart RNA were constructed for both the reference and the genes of interest. For quantification, the relative quantities of the above genes were normalized against the reference gene 36B4.

Mitochondrial DNA levels were measured by real-time quantitative PCR. DNA was extracted from snap-frozen crushed LV heart tissue from GAMT-/- (n=5) and WT litter mates (n=6 ) using the QIAamp DNA micro kit (Qiagen, UK) with RNase-A treatment. DNA was quantified with Quant-iT dsDNA HS assay kit (Invitrogen) and 10ng DNA was used for PCRs performed in triplicate with TaqMan Universal PCR Master Mix (Applied Biosystems) on the Rotor-Gene system (Corbett Research Ltd, Qiagen,UK). Primers and TaqMan probe used to amplify BL/6 mitochondrial DNA (Supplemental Table 4). Mouse ACTB (Actin, beta) Endogenous Control (FAM Dye/MGB probe, Non-primer limited) (Applied Biosystems) was used for nuclear DNA.

| **Gene Symbol** | **Sense** | **Antisense** |
| --- | --- | --- |
| Biogenesis | | |
| *Sirt1* | 5’CCTTCAGTGTCATGGTTCCTT3’ | 5’GCAAGTGGCTCATCAGCT3’ |
| *Pgc1α* | 5’GGGGGAGCCGTCTCTACTTAAGAAGCT3’ | 5’GGGGGGTTTGTTCTGATCCT3’ |
| *Pgc1β* | 5’GGGGCTGACACGCAGGGTGGGGA3’ | 5’GGGGCCGGGCACCACTGCA3’ |
| *Nrf1* | 5’TGACCCAGGCTCAGCTTC3’ | 5’ATTGGCCCATGGGATATCTT3’ |
| *Nfe212* | 5’TGACCATGAGTCGCTTGCC3’ | 5’CCATGTCCTGCTCTATGCTGC3’ |
| *Tfam* | 5’GGGGCCCTCGTCTATCAGTCTTGTCTG3’ | 5’GGGGGCATCTGGGTGTTTAGCTTTA3’ |
| *RIP140* | 5’ACTTCCCGCTGCAGAAACTA3’ | 5’GCGTTTCCCAGAAGTCCATA3’ |
| Metabolic substrate utilisation | | |
| *Glut1* | 5’CTTGTGGCCTCTGCTGCT3’ | 5’GCTTCTTCAGCACACTCTTGG3’ |
| *Glut4* | 5’GGTTGCCCAGGTGCTGGG3’ | 5’GGCAGGCCCCTCCAGG3’ |
| *Pdk4* | 5’GGGGGCGGCAAGAGCTGCCCG3’ | 5’GGGGCTCTGGATATACCAGCTCTTC3’ |
| *Cpt1β* | 5’GGAAAGGTATGGCCACTT3’ | 5’CCCGTGGTAGGAGAGCAG3’ |
| *Pparα* | 5’GGGGGTCCCTTATCTGAAGAA3’ | 5’GCTGGAGAGAGGGTGTCTGTG3’ |
| Mitochondrial Fission and Fusion | | |
| *Drp1* | 5′GCGCTGATCCCGCGTCAT 3′ | 5’CCGCACCCACTGTGTTGA 3′ |
| *Fis1* | 5′GCCCCTGCTACTGGACCAT | 5′CCCTGAAAGCCTCACACTAAGG 3′ |
| *Mfn1* | 5’CTGTTAATCAGCTGGCCCATG3’ | 5’GTACCTGGGCTGTCTAC3’ |
| *Mfn2* | 5’GCTTCCTTGAAGACACCC3’ | 5’TGCCGCCTGGCCAGCACTT3’ |
| *Opa1* | 5’GGGGTGTCAACACATGTG3’ | 5’GGCCAACCTCGACTGCT3’ |
| *Mffb* | 5’GGGGGTGGTTACAGGAAATAA3’ | 5’TGTGAGCACGCGTGGT3’ |
| Mitophagy | | |
| *Pink* | 5’cgtggagaca aagctgcaga3’ | 5’cacagagcca tcatggggct3’ |
| *Parkin* | 5’cccggtgacc atgatagtgt3’ | 5’aaccacttcc ttgagctgca3’ |
| *Mitochondrial DNA Experiments* | | |
| Mouse mtDNAF | 5’ GGGATAACAGCGCAATCCTATT 3’ |  |
| Mouse mtDNAR | 5’ CCAACATCGAGGTCGTAAACC 3’ |  |
| mouse mtDNA MGB probe | 5’ VIC-AGAGTTCATATCGACAATTA-MGBNFQ 3’ |  |
| mtDNAPCR template (HPLC purified) Positive control | 5’GGGATAACAGCGCAATCCTATTTAAGAGTTCATATCGACAATTAGGGTTTACGACCTCGATGTTGG 3’ |  |

**Supplementary Table 3. Primer Details**

**References**

Aksentijevic, D., Zervou, S., Faller, K.M., Mcandrew, D.J., Schneider, J.E., Neubauer, S., and Lygate, C.A. (2014). Myocardial creatine levels do not influence response to acute oxidative stress in isolated perfused heart. *PLoS ONE* 9**,** e109021.

Das, A.M., and Harris, D.A. (1990). Regulation of the mitochondrial ATP synthase in intact rat cardiomyocytes. *Biochem J* 266**,** 355-361.

Di Lisa, F., Kaludercic, N., Carpi, A., Menabo, R., and Giorgio, M. (2009). Mitochondrial pathways for ROS formation and myocardial injury: the relevance of p66(Shc) and monoamine oxidase. *Basic research in cardiology* 104**,** 131-139.

Divald, A., Kivity, S., Wang, P., Hochhauser, E., Roberts, B., Teichberg, S., Gomes, A.V., and Powell, S.R. (2010). Myocardial ischemic preconditioning preserves postischemic function of the 26S proteasome through diminished oxidative damage to 19S regulatory particle subunits. *Circ Res* 106**,** 1829-1838.

Divald, A., and Powell, S.R. (2006). Proteasome mediates removal of proteins oxidized during myocardial ischemia. *Free Radic Biol Med* 40**,** 156-164.

Faller, K.M.E., Atzler, D., Mcandrew, D.J., Zervou, S., Whittington, H.J., Simon, J.N., Aksentijevic, D., Ten Hove, M., Choe, C.U., Isbrandt, D., Casadei, B., Schneider, J.E., Neubauer, S., and Lygate, C.A. (2018). Impaired cardiac contractile function in arginine:glycine amidinotransferase knockout mice devoid of creatine is rescued by homoarginine but not creatine. *Cardiovasc Res* 114**,** 417-430.

Mazzio, E.A., and Soliman, K.F. (2004). Glioma cell antioxidant capacity relative to reactive oxygen species produced by dopamine. *Journal of applied toxicology : JAT* 24**,** 99-106.

Palmer, J.W., Tandler, B., and Hoppel, C.L. (1977). Biochemical properties of subsarcolemmal and interfibrillar mitochondria isolated from rat cardiac muscle. *J Biol Chem* 252**,** 8731-8739.

Riederer, P., Lachenmayer, L., and Laux, G. (2004). Clinical applications of MAO-inhibitors. *Current medicinal chemistry* 11**,** 2033-2043.

Rosing, J., Harris, D.A., Kemp, A., Jr., and Slater, E.C. (1975). Nucleotide-binding properties of native and cold-treated mitochondrial ATPase. *Biochim Biophys Acta* 376**,** 13-26.

Scholz, T.D., and Balaban, R.S. (1994). Mitochondrial F1-ATPase activity of canine myocardium: effects of hypoxia and stimulation. *Am J Physiol* 266**,** H2396-2403.

Shirendeb, U.P., Calkins, M.J., Manczak, M., Anekonda, V., Dufour, B., Mcbride, J.L., Mao, P., and Reddy, P.H. (2012). Mutant huntingtin's interaction with mitochondrial protein Drp1 impairs mitochondrial biogenesis and causes defective axonal transport and synaptic degeneration in Huntington's disease. *Hum Mol Genet* 21**,** 406-420.

Ten Hove, M., Makinen, K., Sebag-Montefiore, L., Hunyor, I., Fischer, A., Wallis, J., Isbrandt, D., Lygate, C., and Neubauer, S. (2008). Creatine uptake in mouse hearts with genetically altered creatine levels. *J Mol Cell Cardiol* 45**,** 453-459.

Weibel, E.R., Kistler, G.S., and Scherle, W.F. (1966). Practical stereological methods for morphometric cytology. *J Cell Biol* 30**,** 23-38.

Youdim, M.B., Edmondson, D., and Tipton, K.F. (2006). The therapeutic potential of monoamine oxidase inhibitors. *Nature reviews. Neuroscience* 7**,** 295-309.
